# Supplementary material for: Production of hydrogen from offshore wind in China and cost-competitive supply to Japan
Source: Nat Commun. 2021 Nov 29;12:6953. doi: 10.1038/s41467-021-27214-7 (PMC8630012; doi:10.1038/s41467-021-27214-7)
Supplement: Supplementary file 1 — Supplementary Information [file 41467_2021_27214_MOESM1_ESM.pdf]

Supplementary Information for

Production of Hydrogen from Offshore Wind in China and Cost-competitive Supply to Japan

Shaojie Song, Haiyang Lin*, Peter Sherman, Xi Yang, Chris P. Nielsen, Xinyu Chen, Michael B. McElroy*

Correspondence to: haiyanglin@seas.harvard.edu (H.L.); mbm@seas.harvard.edu (M.B.M.).

These authors contributed equally: Shaojie Song, Haiyang Lin.

This PDF file includes:

Mathematical Methods

Figures S1 to S5

Tables S1 to S7

Mathematical Methods

The potential costs and related quantities of hydrogen that could be supplied to Japan are obtained based on a least-cost hydrogen delivery model optimizing jointly investment decisions and hourly operations accounting for the volatility of offshore wind power. The model allows for grid by grid analysis of offshore wind deployment, for operation of electrolysis equipment, for hydrogen conversion, for energy storage, and for related overseas transportation.

The optimization model is designed to minimize the overall system costs (C_{tot}), which consist of three components: amortized investment costs (C_{inv}), fixed O&M (Operations and Maintenance) costs (C_{om}), and annual operational costs (C_{op}). C_{op} include the costs for materials in hydrogen conversion (C_{mt}), freshwater (C_{wt}), shipping fuel (C_{shf}), heating source (C_{ht}), and grid electricity (C_{gd}), and are offset by income from selling by-product oxygen (C_{o2}). The objective of minimization is shown in Eq. S1, and the expressions for the three cost components are provided in Eqs. S2 and S3.

$$\text{Min } C_{tot} = C_{inv} + C_{om} + \left(C_{mt} + C_{wt} + C_{shf} + C_{ht} + C_{gd} - C_{o2}\right) \tag{S1}$$

$$\begin{cases} C_{inv} = \sum_{x=wd}^{rl}\left(\alpha_x I_x i \frac{(1+i)^{T_x}}{(1+i)^{T_x}-1}\right) \\ C_{om} = \sum_{x=wd}^{rl}\left(\alpha_x \beta_x I_x\right) \\ x = [wd; ec; cop; gs; cov; cs; cspu; bs; sh; shpu; rl] \end{cases} \tag{S2}$$

$$\begin{cases} C_{gd} = \gamma_{gd,cn} \sum_{t=1}^{8760} P_{CN}^{t} + \gamma_{gd,jp} \sum_{t=1}^{8760} P_{JP}^{t} \\ C_{wt} = \gamma_{wt} r_{wt} \sum_{t=1}^{8760} H_{ec}^{t} \\ C_{o2} = \gamma_{o2} r_{o2} \sum_{t=1}^{8760} H_{ec}^{t} \\ C_{mt} = \gamma_{mt} \sum_{t=1}^{8760} H_{cov}^{t} \\ C_{shf} = \gamma_f r_f I_{sh} n_{trip} t_{sh} \\ C_{ht} = \gamma_{ht} r_{ht} \sum_{t=1}^{8760} H_{rl}^{t} \end{cases} \tag{S3}$$

where α_x, β_x, I_x, and T_x denote the capital costs, O&M fractions, installed capacities, and lifetimes for wind turbines (x = wd), electrolyzers (x = ec), compressors (x = cop), geological storage (x = gs), conversion plants (x = cov), carrier storage (x = cs), carrier pump (x = cspu), buffer storage (x = bs), ships (x = sh), ship pump (x = shpu), and hydrogen releasing plants (x = rl), respectively. The symbol i represents the interest rate. $\gamma_{gd,cn}$ and $\gamma_{gd,jp}$ denote the unit prices for grid electricity in China and Japan. γ_{wt}, γ_{o2}, γ_{mt}, γ_{shf}, and γ_{ht} represent the unit prices of freshwater, oxygen, material for hydrogen conversion, shipping fuel, and heating sources, respectively. r_{wt}, r_{o2}, r_{shf}, and r_{ht} define the demands for freshwater, oxygen, shipping fuel, and heating sources, respectively. P^t_{CN} and P^t_{JP} represent the amount of integrated grid power required at the Chinese and Japanese ports at time t, respectively. The hydrogen or hydrogen equivalent output from the electrolysis system, conversion plant, and the releasing plant at time t are denoted by H^t_{ec}, H^t_{cov}, and H^t_{rl}, respectively.

The annual number of round trips per ship, n_{trip}, is calculated using the annual operation days for the ship (*Aod*), the sailing time for each trip (t_{sh}), and the time for loading or unloading at the ports (t_{load}):

$$n_{trip} = \frac{Aod}{2(t_{sh} + t_{load})} \tag{S4}$$

The model considers five types of constraints including renewable power generation, performances of devices, operations for energy storage and power and hydrogen flow balances.

The integrated wind power at time t, P^t_{wd}, is constrained by the installed wind capacity, I_{wd}, and the capacity factor at time t, cf^t:

$$0 \leq P^t_{wd} \leq I_{wd} cf^t \tag{S5}$$

The performance of devices includes their energy conversion and consumption, upper and lower load limitation, and the ramping constraint:

$$P_{ec}^{t} = \frac{H_{ec}^{t}}{e_{ec}} \tag{S6}$$

$$P_{cop}^{t} = H_{cop}^{t} r_{cop} \tag{S7}$$

$$P_{cov}^{t} = H_{cov}^{t} r_{cov} \tag{S8}$$

$$P_{cspu}^{t} = H_{cspu}^{t} r_{pu} \tag{S9}$$

$$P_{shpu,l}^{t} = H_{shpu,l}^{t} r_{pu} \tag{S10}$$

$$P_{shpu,u}^{t} = H_{shpu,u}^{t} r_{pu} \tag{S11}$$

$$0 \leq P_{ec}^{t} \leq I_{ec} \tag{S12}$$

$$0 \leq H_{cop}^{t} \leq I_{cop} \tag{S13}$$

$$0.2 I_{cov} \leq H_{cov}^{t} \leq I_{cov} \tag{S14}$$

$$-0.06 I_{cov} \leq H_{cov}^{t+1} - H_{cov}^{t} \leq 0.06 I_{cov} \tag{S15}$$

where P_{ec}^{t}, P_{cop}^{t}, P_{cov}^{t}, P_{cspu}^{t}, $P_{shpu,l}^{t}$, and $P_{shpu,u}^{t}$ indicate the power demand for electrolysis, hydrogen compression, conversion, carrier pumping and loading and unloading with pump at time t, respectively. H_{cop}^{t}, H_{cspu}^{t}, $H_{shpu,l}^{t}$, and $H_{shpu,u}^{t}$ represent the hydrogen flow of compression, carrier pumping and loading and unloading with pump at time t, respectively. e_{ec} is the power to hydrogen efficiency (lower heating value). The power consumed in compression, conversion, and pumping 1 kg of hydrogen or hydrogen equivalent is represented by r_{cop}, r_{cov}, and r_{pu}, respectively. The minimum load and ramping constraint for conversion plants are assumed to be 20% and 6%, respectively[1].

Constraints relating to operations for energy storage are considered from both energy and power perspectives. The hydrogen charging ($H^t_{gs,cha}$) and discharging ($H^t_{gs,dis}$) for geological storage or storage in gaseous hydrogen tanks are constrained by the installed capacity of supporting compressors. The carrier storage (liquid hydrogen, MCH, and ammonia) is charged ($H^t_{cs,cha}$) and discharged ($H^t_{cs,dis}$) by pumping. The stored amount of hydrogen (S^t_{gs}) and products (S^t_{cs}) are constrained by the capacity of the applicable storage facility.

$$\begin{cases} 0 \le H^t_{gs,cha} \le I_{cop} \\ 0 \le H^t_{gs,dis} \le I_{cop} \\ 0 \le S^t_{gs} \le I_{gs} \\ S^{t+1}_{gs} = S^t_{gs} + e_{gs,cha} H^t_{gs,cha} - \dfrac{H^t_{gs,dis}}{e_{gs,dis}} - e_{gs,self} S^t_{gs} \\ S^1_{gs} = S^{8760}_{gs} \end{cases} \quad \text{(S16)}$$

$$\begin{cases} 0 \le H^t_{cs,cha} \le I_{cspu} \\ 0 \le H^t_{cs,dis} \le I_{cspu} \\ 0 \le S^t_{cs} \le I_{cs} \\ S^{t+1}_{cs} = S^t_{cs} + e_{cs,cha} H^t_{cs,cha} - \dfrac{H^t_{cs,dis}}{e_{cs,dis}} - e_{cs,self} S^t_{cs} \\ S^1_{cs} = S^{8760}_{cs} \end{cases} \quad \text{(S17)}$$

where $e_{y,cha}$, $e_{y,dis}$, and $e_{y,self}$ represent the charging efficiency, discharging efficiency and self-discharge rate for gaseous hydrogen storage (y = gs) and carrier storage (y = cs), respectively.

The power demand for individual applicable technologies and power generation are balanced for every time interval.

$$P^t_{ec} + P^t_{cop} + P^t_{cov} + P^t_{cspu} + P^t_{shpu,l} = P^t_{wd} + P^t_{CN} \quad \text{(S18)}$$

$$P^t_{rl} + P^t_{shpu,u} = P^t_{JP} \quad \text{(S19)}$$

The constraints on hydrogen flow consider the loss rate for hydrogen conditioning implicated in delivery of 1 million tons (Mt), H_{tot}, to Japan annually:

$$H_{ec}^{t} + H_{gs,dis}^{t} = H_{gs,cha}^{t} + H_{cov}^{t} \tag{S20}$$

$$H_{cop}^{t} = H_{gs,cha}^{t} \tag{S21}$$

$$H_{cov}^{t} + H_{cs,dis}^{t} = H_{cs,cha}^{t} + \frac{I_{sh}H_{sh}n_{trip}}{8760(1 - e_{bs})\left(1 - e_{pu}\right)} \tag{S22}$$

$$H_{cspu}^{t} = H_{cs,dis}^{t} + H_{cs,cha}^{t} \tag{S23}$$

$$I_{bs} = t_{bs}\frac{I_{sh}H_{sh}n_{trip}}{8760(1 - e_{bs})(1 - e_{pu})} \tag{S24}$$

$$I_{sh}H_{sh}n_{trip} = \frac{\sum_{t=1}^{8760} H_{rl}^{t}}{(1 - e_{pu})(1 - e_{sh})(1 - e_{rl})} \tag{S25}$$

$$I_{pu} = \frac{I_{sh}H_{sh}}{t_{load}(1 - e_{pu})} \tag{S26}$$

$$\sum_{t=1}^{8760} H_{pu}^{t} = \frac{\sum_{t=1}^{8760} H_{rl}^{t}}{(1 - e_{pu})(1 - e_{rl})} + \frac{\sum_{t=1}^{8760} H_{rl}^{t}}{(1 - e_{pu})^{2}(1 - e_{sh})(1 - e_{rl})} \tag{S27}$$

$$\sum_{t=1}^{8760} H_{rl}^{t} = H_{tot} \tag{S28}$$

where e_{rl}, e_{pu}, e_{bs}, e_{sh}, and e_{cov} indicate the loss rates for hydrogen during different conditioning processes. H_{sh} is the payload of each ship. t_{bs} represents the time for buffer storage.

With the above formulation of the optimization model, the levelized cost of hydrogen (LCOH) and delivered quantity of hydrogen (H_d) for the studied grid (0.1° longitude by 0.1° latitude) can be derived according to the physical offshore wind capacity (I_{off}):

$$LCOH = \frac{C_{tot}}{H_{tot}} \tag{S29}$$

$$H_d = \frac{I_{off}}{I_{wd}} H_{tot} \tag{S30}$$

We arranged spatial LCOH values from smallest to largest on a national and province-by-province basis and then calculated the weighted average LCOH for cumulative hydrogen quantities, integrating them to obtain estimates of relevant cost curves (Figure 1) and cost competitive capacities (Table 1).

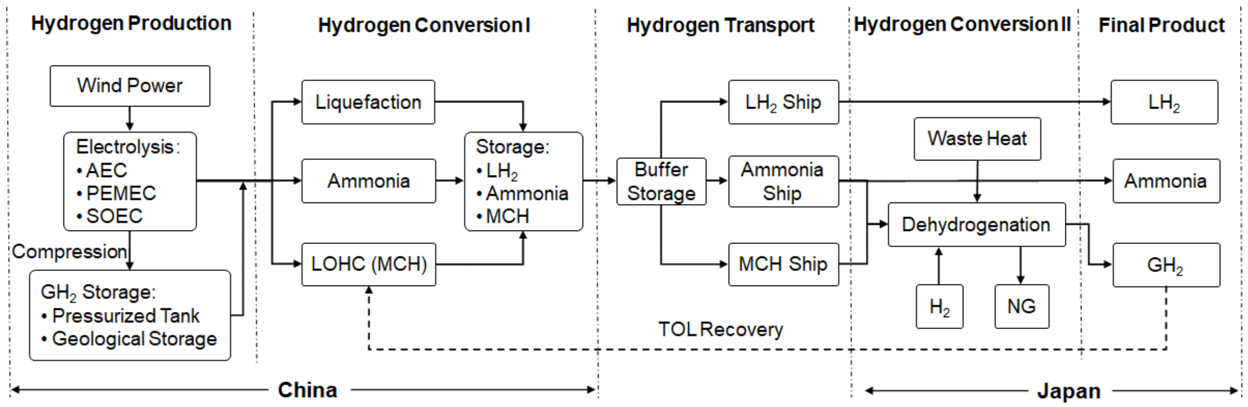

Figure S1. China-Japan hydrogen supply chain scheme. (AEC: alkaline electrolyzer cells, PEMEC: proton exchange membrane electrolyzer cells, SOEC: solid oxide electrolyzer cells, GH_2: gaseous hydrogen, LH_2: liquid hydrogen, LOHC: liquid organic hydrogen carrier, MCH: methylcyclohexane, NG: natural gas).

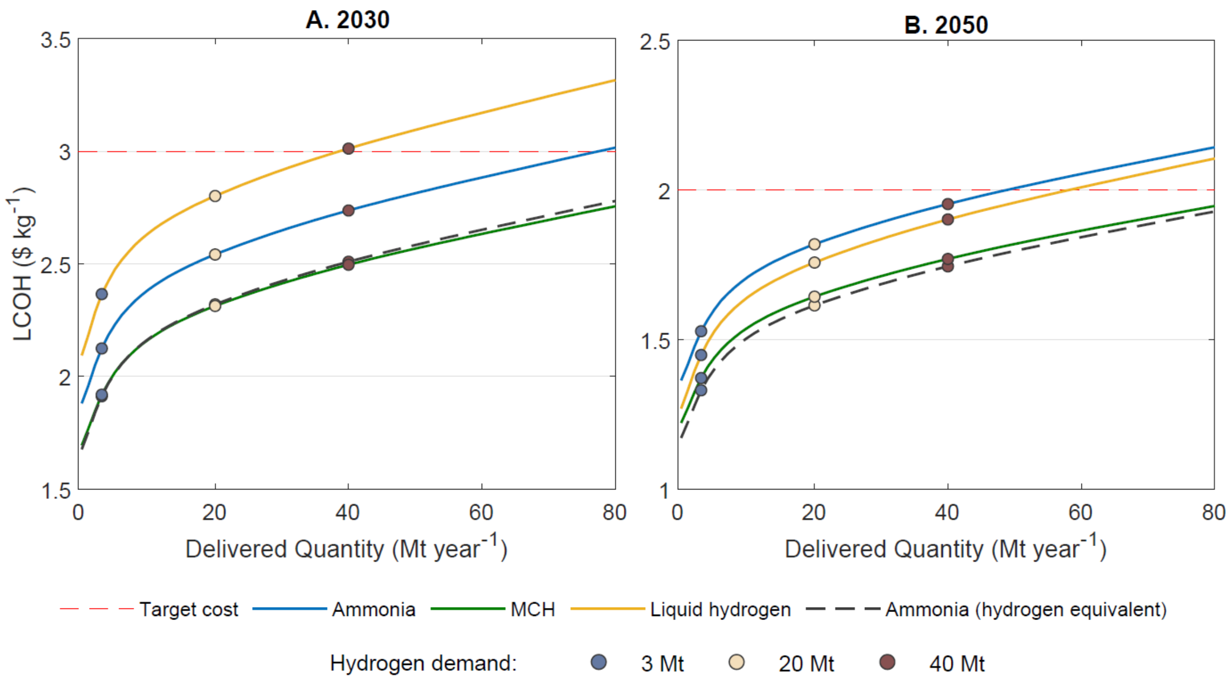

Figure S2. Supply curves for hydrogen delivered to Japan with the application of geological storage in China. (A) and (B) are for 2030 and 2050, respectively. Alkaline electrolyzer cells (AEC) is used for hydrogen production. Dehydrogenation heat source is waste heat. The levelized cost of hydrogen (LCOH) is calculated as the weighted average cost for the cumulative quantity of hydrogen. MCH represents the hydrogen transport mechanism as in methylcyclohexane.

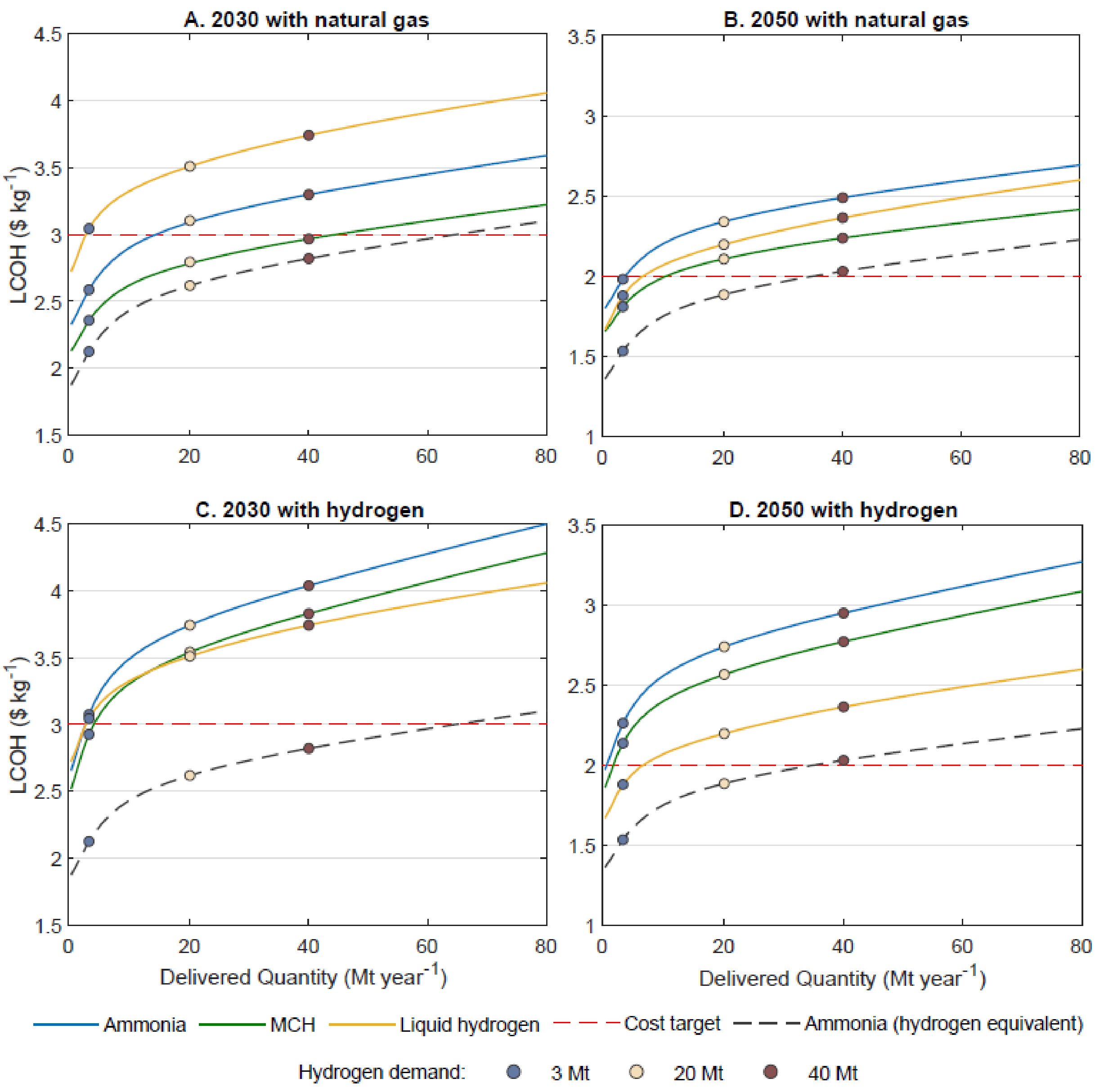

Figure S3. Supply curves for hydrogen delivered to Japan with natural gas (A and B) and hydrogen (C and D) as dehydrogenation heat sources. (A) and (C) are for 2030 and (B) and (D) are for 2050, respectively. Alkaline electrolyzer cells (AEC) is used for hydrogen production. Pressurized tanks are the taken option for gaseous hydrogen storage. The levelized cost of hydrogen (LCOH) is calculated as the weighted average cost for the cumulative quantity of hydrogen. MCH represents the hydrogen transport mechanism as in methylcyclohexane.

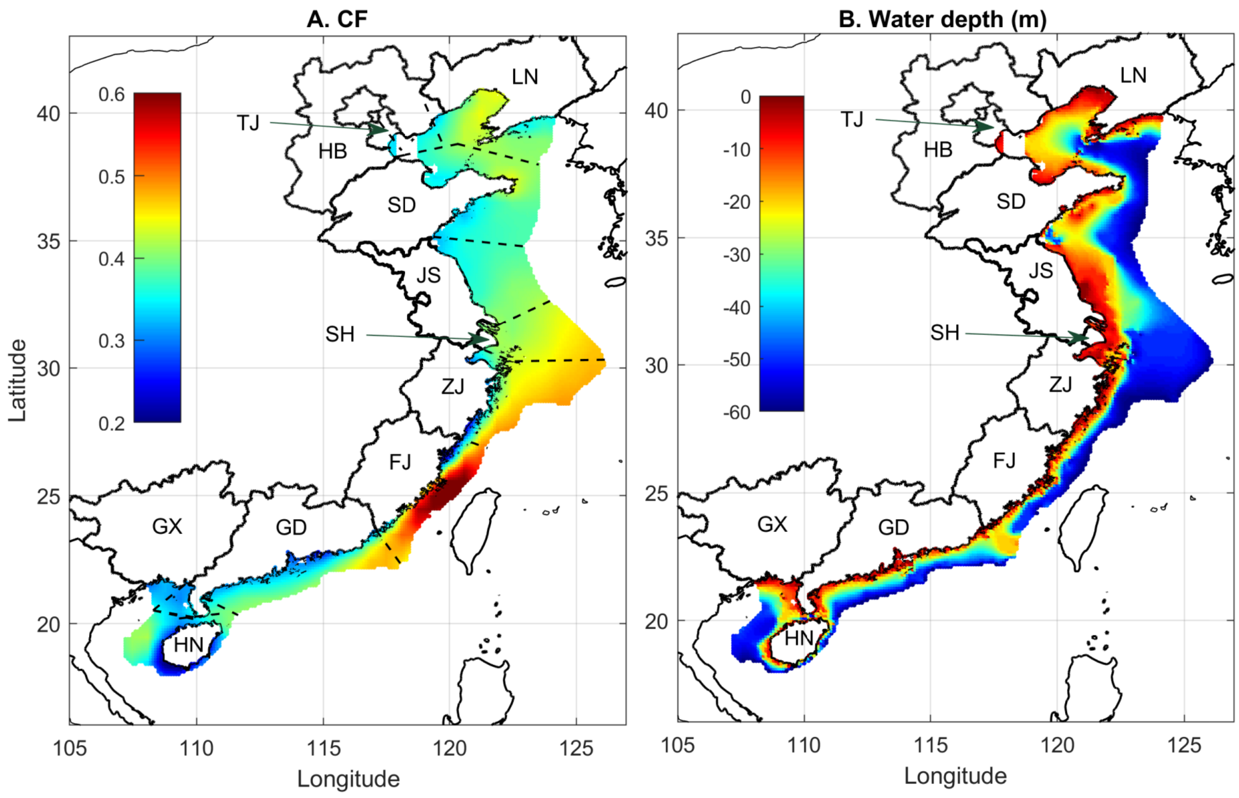

Figure S4. Capacity factors (CFs) and water depths. Spatial distributions of the mean (A) CFs over 1990–2019 and the current (B) water depth (m) for the filtered region. The dashed black lines denote offshore wind areas that are in closest proximity to particular provinces. LN, TJ, HB, SD, JS, SH, ZJ, FJ, GD, GX, and HN are the abbreviations for Liaoning, Tianjin, Hebei, Shandong, Jiangsu, Shanghai, Zhejiang, Fujian, Guangdong, Guangxi, and Hainan, respectively.

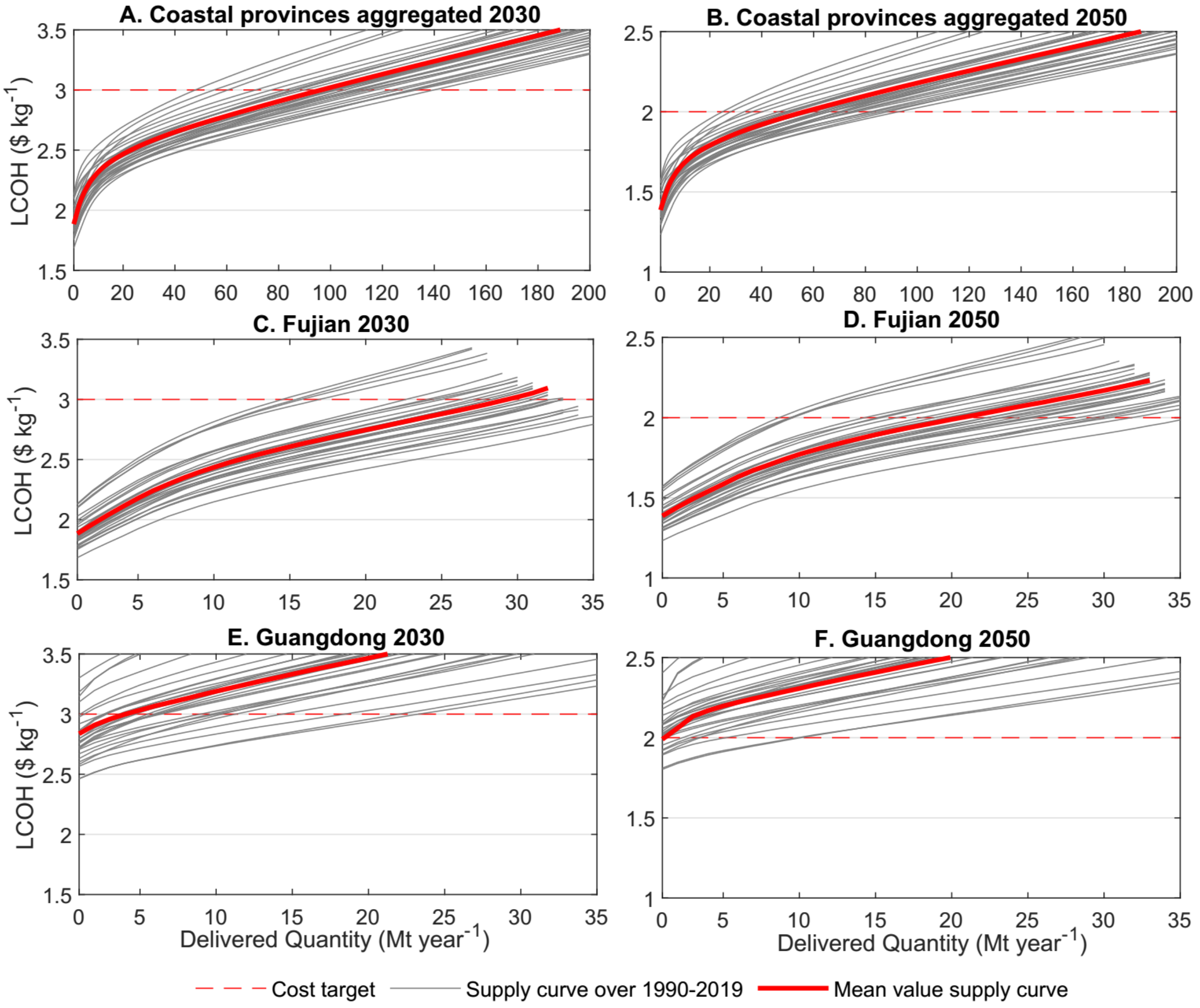

Figure S5. Hydrogen supply curves as in MCH form over 1990-2019 and the mean value curve for 2030 (A, C and E) and 2050 (B, D and F) scenarios. The levelized cost of hydrogen (LCOH) is calculated as the weighted average cost for the cumulative quantity of hydrogen. Heating source for hydrogen releasing from (methylcyclohexane) MCH is waste heat. Alkaline electrolyzer cells (AEC) is used for hydrogen production. Pressurized tanks are the taken option for gaseous hydrogen storage.

Table S1. Techno-economic characteristics of different water electrolysis technologies.

	AEC[c]		PEMEC[d]		SOEC[e]		Reference
	2030	2050	2030	2050	2030	2050	
Capital cost ($ kW^{-1})	135	98	650	200	800	500	[2,3]
Electrical efficiency (%, LHV[a])	65	70	63	67	77	77	[2]
Annual O&M[b] cost (% of capital cost)	2		4		3		[4]
System lifetime (year)	30		20		20		[4]

[a] LHV = lower heating value.

[b] O&M = operation and maintenance.

[c] AEC= alkaline electrolyzer cells.

[d] PEMEC= proton exchange membrane electrolyzer cells.

[e] SOEC= solid oxide electrolyzer cells.

Table S2. Techno-economic data for hydrogen conversion.

	Compression		Ammonia production		MCH[e] production	
	2030	2050	2030	2050	2030	2050
Capital cost (k\$ kg^{-1} h^{-1})[a]	0.41	0.36	16	14.5	1.05	0.85
Unit capacity (ton day^{-1})	200	200	285	285	210	435
Electricity demand (kWh kg^{-1})	0.84	0.84	2.65	2.65	1.5	1.5
Material cost (\$ kg^{-1})[b]	0		0.002		0.013	
Scale factor[c]	0.66		0.7		0.7	
Loss (%)	0.5		3		1	
Annual O&M[d] cost (% of capital cost)	2		4		4	
System lifetime (year)	15		20		20	
Reference	[5]		[6,7]		[7,8]	
	Liquefaction		Ammonia cracking		MCH cracking	
	2030	2050	2030	2050	2030	2050
Capital cost (k\$ kg^{-1} h^{-1})	25	18	1	0.9	0.85	0.7
Unit capacity (ton day^{-1})	300	300	430	430	405	405
Electricity demand (kWh kg^{-1})	6.76	6	1.51	1.51	1.52	1.52
Heat demand (kWh kg^{-1})	0		10		13	
Scale factor	0.66		0.7		0.7	
Loss (%)	1.65		2		2	
Annual O&M cost (% of capital cost)	4		4		4	
System lifetime (year)	20		20		20	
Reference	[5]		[6,7]		[7,8]	

Notes: All the parameters are hydrogen or hydrogen equivalent based value.

[a] Capital cost is the levelized investment for plants at their unit capacities.

[b] Material cost includes carrier cost and catalyst cost.

[c] Scale factor is only used when designed system capacity is smaller than unit capacity.

[d] O&M = operation and maintenance.

[e] MCH= methylcyclohexane.

Table S3. Techno-economic data for storage systems.

	Salt Cavern[a]		Pressurized Tank		Liquid H_2		Ammonia[b]		MCH[c]	
	2030	2050	2030	2050	2030	2050	2030	2050	2030	2050
Capital cost ($ kg^{-1})	5.7	4.5	500	450	26	18	2.6	2.6	4.6	4.6
Loss (%)	0		0		0.1 day^{-1}		0		0	
System lifetime (year)	30		30		20		20		20	
Annual O&M[d] cost [% of capital cost]	2									
Reference	5		5		5,7		9		8	

Notes: All the parameters are hydrogen or hydrogen equivalent based value.
[a] The cost is levelized for a 1100-ton geologic storage system.
[b] Cost converted from market price for 10000-gallon ammonia storage tanks.
[c] Cost converted from oil tank price, MCH is the abbreviation of methylcyclohexane.
[d] O&M = operation and maintenance.

Table S4. Techno-economic data for hydrogen transportation.

For Ship	Liquid H_2		Ammonia		MCH^b	
	**2030**	**2050**	**2030**	**2050**	**2030**	**2050**
Capital cost (M$ ship^{-1})	398	213	52	52	35	35
Unit capacity (m^3)	160000		38000		91000	
Fuel consumption (ton day^{-1})	45		50		80	
Loss (%)	0.2 day^{-1}		0		0	
Average speed (knot)			15			
Annual operation days			329			
Load/unload time (hour)			48			
Annual O&M[a] cost [% of Capital cost]			4			
System lifetime (year)			25			
Reference	[7]		[7,10]		[7,10]	

For Pump	Liquid H_2		Ammonia		MCH	
	**2030**	**2050**	**2030**	**2050**	**2030**	**2050**
Capital cost ($ kg^{-1} h^{-1})	43		11		24	
Electricity demand (kWh kg^{-1})	0.001		0.012		0.032	
Loss (%)	0.2 day^{-1}		0		0	
Annual O&M cost [% of Capital cost]			4			
System lifetime (year)			10			
Reference	[5]		[11]		[11]	

Notes: All the parameters are hydrogen or hydrogen equivalent based value. Fuel price: $430 ton^{-1}.
[a] O&M = operation and maintenance.
[b] MCH= methylcyclohexane.

Table S5. Shipping lines between China coastal cities and Kobe, Japan (ref. [12]).

China city	Japan city	Distance (km)	Trip time (one way)
Dalian, Liaoning		1488.7	2 days 5 hours
Tianjin		1802.1	2 days 16 hours
Huanghua, Hebei		1764.2	2 days 15 hours
Qingdao, Shandong		1406.9	2 days 2 hours
Lianyungang, Jiangsu		1465.5	2 days 4 hours
Shanghai	Kobe	1331.2	1 day 23 hours
Jiaxing, Zhejiang		1401.9	2 days 2 hours
Xiamen, Fujian		2040.0	3 days 1 hour
Bao'An, Guangdong		2556.7	3 days 10 hours
Qinzhou, Guangxi		3219.9	4 days 19 hours
Macun, Hainan		3007.6	4 days 12 hours

Table S6. Levelized cost of delivered hydrogen. Alkaline electrolyzer cells (AEC) is used for hydrogen production. Pressurized tanks are the taken option for gaseous hydrogen storage. MCH represents the hydrogen transport mechanism as in methylcyclohexane.

Wind system cost	Supply chain	2030 (3Mt) $ kg^{-1}	2050 (20Mt) $ kg^{-1}
High-cost scenario	MCH (waste heat)	2.54	2.53
	Ammonia (waste heat)	2.87	2.89
	Liquid H_2	3.57	3.03
	MCH (natural gas)	2.98	2.98
	Ammonia (natural gas)	3.22	3.24
	MCH (hydrogen)	3.64	3.64
	Ammonia (hydrogen)	3.79	3.81
	Ammonia	2.65	2.66
Moderate-cost scenario	MCH (waste heat)	2.04	1.79
	Ammonia (waste heat)	2.34	2.10
	Liquid H_2	3.04	2.20
	MCH (natural gas)	2.49	2.24
	Ammonia (natural gas)	2.69	2.44
	MCH (hydrogen)	2.93	2.57
	Ammonia (hydrogen)	3.07	2.74
	Ammonia	2.13	1.89
Low-cost scenario	MCH (waste heat)	1.69	1.40
	Ammonia (waste heat)	1.99	1.68
	Liquid H_2	2.58	1.72
	MCH (natural gas)	2.14	1.84
	Ammonia (natural gas)	2.33	2.02
	MCH (hydrogen)	2.42	2.00
	Ammonia (hydrogen)	2.59	2.19
	Ammonia	1.77	1.48

Table S7. Levelized cost of delivered hydrogen in the moderate wind cost scenario. Pressurized tanks are the taken option for gaseous hydrogen storage. MCH represents the hydrogen transport mechanism as in methylcyclohexane. PEMEC and SOEC are the abbreviations for proton exchange membrane electrolyzer cells and solid oxide electrolyzer cells, respectively.

Electrolyzer	Supply chain	2030 (3Mt) $ kg^{-1}	2050 (20Mt) $ kg^{-1}
PEMEC	MCH (waste heat)	2.85	2.03
	Ammonia (waste heat)	3.17	2.35
	Liquid H_2	3.83	2.43
	MCH (natural gas)	3.30	2.48
	Ammonia (natural gas)	3.52	2.69
	MCH (hydrogen)	4.07	2.92
	Ammonia (hydrogen)	4.17	3.08
	Ammonia	2.94	2.13
SOEC	MCH (waste heat)	2.43	2.13
	Ammonia (waste heat)	2.74	2.44
	Liquid H_2	3.37	2.53
	MCH (natural gas)	2.88	2.58
	Ammonia (natural gas)	3.09	2.78
	MCH (hydrogen)	3.48	3.05
	Ammonia (hydrogen)	3.59	3.20
	Ammonia	2.52	2.22

Supplementary References:

1. Nayak-Luke, R., Bañares-Alcántara, R. & Wilkinson, I. "Green" Ammonia: Impact of Renewable Energy Intermittency on Plant Sizing and Levelized Cost of Ammonia. *Ind. Eng. Chem. Res.* **57**, 14607-14616, doi:10.1021/acs.iecr.8b02447 (2018).

2. International Energy Agency (IEA). *The Future of Hydrogen*, <www.iea.org/reports/the-future-of-hydrogen> (2019).

3. Bloomberg Finance L.P. Hydrogen Economy Outlook: Key Messages. (Singapore, 2020).

4. Buttler, A. & Spliethoff, H. Current status of water electrolysis for energy storage, grid balancing and sector coupling via power-to-gas and power-to-liquids: A review. *Renew. Sustain. Energy Rev* **82**, 2440-2454, doi:10.1016/j.rser.2017.09.003 (2018).

5. US DOE. The Fuel Cell Technologies Office Multi-Year Research, Development, and Demonstration Plan. (Technical report: US Department of Energy, 2016).

6. Dias, V., Pochet, M., Contino, F. & Jeanmart, H. Energy and economic costs of chemical storage. *Front. Mech. Eng.* **6**, doi:10.3389/fmech.2020.00021 (2020).

7. New Energy and Industrial Technology Development Organization (NEDO). Advancement of Hydrogen Technologies and Utilization Project (FY2014-FY2015) Final Report. (Tokyo, Japan, 2016).

8. Niermann, M., Drünert, S., Kaltschmitt, M. & Bonhoff, K. Liquid organic hydrogen carriers (LOHCs) – techno-economic analysis of LOHCs in a defined process chain. *Energy Environ. Sci.* **12**, 290-307, doi:10.1039/C8EE02700E (2019).

9. National tank outlet. *Ammonia storage tanks for sale*, <www.ntotank.com/ammonia-storage-tanks> (2021).

10. Niermann, M., Timmerberg, S., Drünert, S. & Kaltschmitt, M. Liquid Organic Hydrogen Carriers and alternatives for international transport of renewable hydrogen. *Renew. Sustain. Energy Rev* **135**, 110171, doi:10.1016/j.rser.2020.110171 (2021).

11. Reuß, M. *et al.* Seasonal storage and alternative carriers: A flexible hydrogen supply chain model. *Appl. Energy* **200**, 290-302, doi:10.1016/j.apenergy.2017.05.050 (2017).

12. SEAPATES by DP WORLD. *Distance & Time tool*, <www.searates.com/services/distances-time/> (2021).
